# Supplementary material for: Anti-topological crystal and non-Abelian liquid in twisted semiconductor bilayers
Source: Nat Commun. 2026 Apr 25;17:3814. doi: 10.1038/s41467-026-70916-z (PMC13121608; doi:10.1038/s41467-026-70916-z)
Supplement: Supplementary file 1 — Supplementary Information [file 41467_2026_70916_MOESM1_ESM.pdf]

Supplemental Material for *Anti-topological crystal and non-Abelian liquid in twisted semiconductor bilayers*

Aidan P. Reddy,[1,*] D. N. Sheng,[2,†] Ahmed Abouelkomsan,[1,‡] Emil J. Bergholtz,[3,§] and Liang Fu[1,¶]

[1]*Department of Physics, Massachusetts Institute of Technology, Cambridge, Massachusetts 02139, USA*
[2]*Department of Physics and Astronomy, California State University Northridge, Northridge, California 91330, USA*
[3]*Department of Physics, Stockholm University, AlbaNova University Center, 106 91 Stockholm, Sweden*

(Dated: February 24, 2026)

CONTENTS

I. Lowest-harmonic model band structure 1

II. Half-filled 1LL in a periodic potential 2

III. Projected structure factor 2

IV. Many-body Berry curvature 4

V. Miscellaneous 5

References 5

I. LOWEST-HARMONIC MODEL BAND STRUCTURE

In Fig. 1, we study the band structure of the lowest harmonic continuum model of Ref. [1] (with model parameters from Ref. [2]). The second band remains rather narrow (<5 meV) throughout the range of twist angles shown. Notably, its Chern number changes from -1 to $+1$ at $\theta \approx 1.9^\circ$ due to a quadratic band inversion with the third miniband at the γ point.

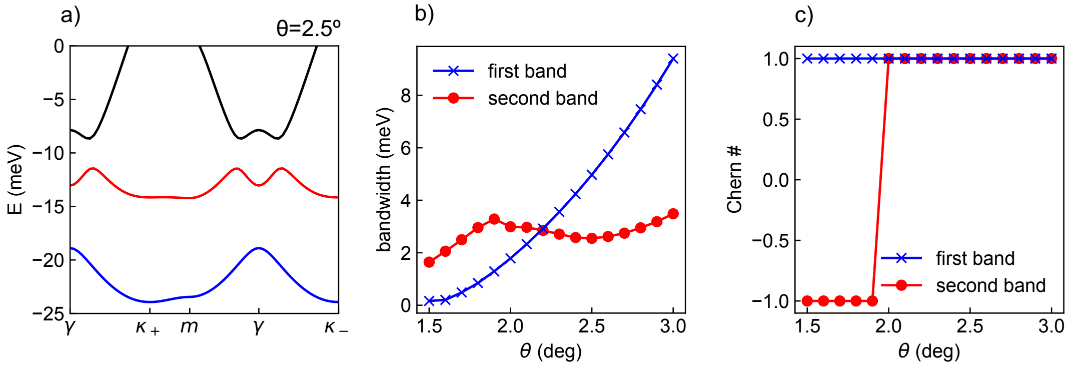

FIG. 1. **Band structure and topology of the lowest-harmonic continuum model.** (a) Band structure of lowest-harmonic continum model at $\theta = 2.5^\circ$. (b) Bandwidths and (c) Chern numbers of first two minibands as a function of twist angle.

* areddy@mit.edu
† donna.sheng1@csun.edu
‡ ahmed95@mit.edu
§ emil.bergholtz@fysik.su.se
¶ liangfu@mit.edu

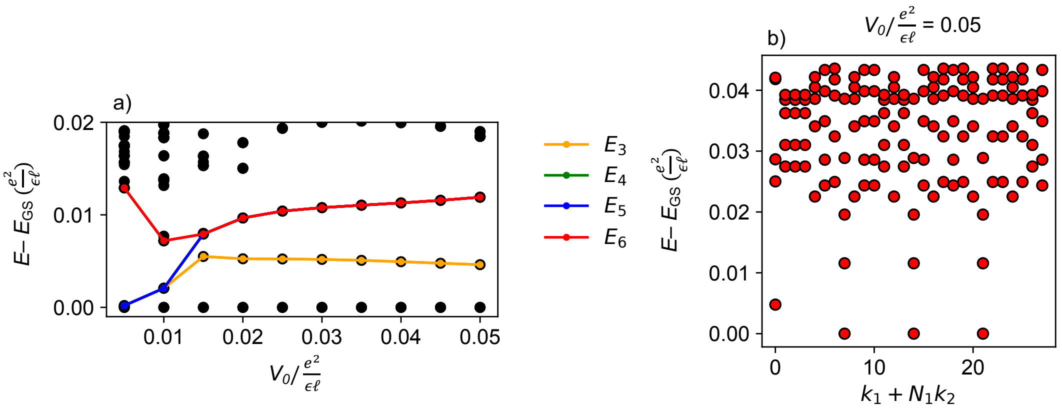

FIG. 2. **Half-filled first-excited Landau level in a periodic potential.** (a) Many-body spectrum of the half-filled 1LL in a weak periodic potential on cluster 28. At small $V_0/\frac{e^2}{\epsilon\ell}$, the system is in a non-Abelian fractional quantum Hall phase with an approximate six-fold ground state degeneracy. As $V_0/\frac{e^2}{\epsilon\ell}$ increases beyond ≈ 0.02, the system transitions to a crystal phase with a fourfold ground state quasidegeneracy and a 2×2 enlarged unit cell. (b) Many-body spectrum as a function of the total crystal momentum at a representative point in the crystal phase.

II. HALF-FILLED 1LL IN A PERIODIC POTENTIAL

As a toy model for the second miniband of $t\mathrm{MoTe}_2$, we study an electron gas in uniform magnetic field and a weak periodic potential. This system is described by the single-particle Hamiltonian Eq. (1) of the main text with $\nabla \times \boldsymbol{A}(\boldsymbol{r}) = -B\hat{z}$ and $V(\boldsymbol{r}) = -2V_0 \sum_{i=1,2,3} \cos(\boldsymbol{g}_i \cdot \boldsymbol{r})$, $\boldsymbol{g}_i = \sqrt{\frac{4\pi}{\sqrt{3}}}\frac{1}{\ell}(\cos\left((i-1)\frac{\pi}{3}\right), \sin\left((i-1)\frac{\pi}{3}\right))$. Here $V(\boldsymbol{r})$ is a lowest-harmonic potential with a triangular Bravais lattice enclosing one flux quantum per unit cell. We consider the limit $\frac{e^2}{\epsilon\ell}, V_0 \ll \hbar\omega_c$ so that Landau level mixing induced by Coulomb interactions or the external potential are negligible.

In Fig. 2, we study the model's phase diagram at $\nu = \frac{3}{2}$ as a function of the strength of the periodic potential relative to interactions, $V_0/\frac{e^2}{\epsilon\ell}$. In the absence of a periodic potential, we recover the well-known non-Abelian fractional quantum Hall state of the half-filled first-excited Landau level (1LL). As the potential strength increases, the ground state transitions to a crystal with a 2×2 enlarged unit cell, as evidenced by the fourfold ground state quasidegeneracy with one ground state at γ and each m point. For $V_0 > 0$ (in which case the minima of the potential form a triangular lattice), we have computed the Chern number (not shown) and find a contribution from the half-full 1LL of 0, leading to $C_{\mathrm{tot}} = 1$ upon accounting for the contribution from the full lowest Landau level. Under a sign change of V_0, the minima of the external potential form a honeycomb lattice and the ground state is obtained by performing a particle-hole conjugation within the 1LL. This particle-hole conjugation changes the contribution to the total Chern number of the 1LL from 0 to 1, leading to $C_{\mathrm{tot}} = 2$. At larger potential strength (not shown), the system has a ground state degeneracy consistent with shell filling, suggesting that it enters a Landau Fermi liquid phase.

In summary, as $V_0/\frac{e^2}{\epsilon\ell}$ sweeps from a small positive to a small negative value, we find the following sequence of phases: 2×2 crystal with $C_{\mathrm{tot}} = 0 \longrightarrow$ non-Abelian fractional quantum Hall state $\longrightarrow 2\times 2$ crystal with $C_{\mathrm{tot}} = 0$. This sequence of phases closely corresponds to the phase diagram we find in the adiabatic model near $\theta = 2.5°$, with $\theta < 2.5°$ corresponding to $V_0 < 0$. However, unlike in the adiabatic model, we do not find an anti-topological crystal phase in this mode

III. PROJECTED STRUCTURE FACTOR

As mentioned in the main text, the 1LL-like character of the second miniband's wavefunctions suppresses finite-size Bragg peaks. In Fig. 3, we show the projected structure factor $s(\boldsymbol{q})$ of the adiabatic model's ground state at several twist angles. In contrast to the modified structure factor $\tilde{s}(\boldsymbol{q})$ shown in Fig. 1 of the main text, $s(\boldsymbol{q})$ does not show incipient Bragg peaks at $\theta = 3°$. Moreover, unlike $\tilde{s}(\boldsymbol{q})$, $s(\boldsymbol{q})$ does not contrast sharply between $\theta = 2.5°$ and $3°$. At $\theta = 2°$, $s(\boldsymbol{q})$ shows Bragg peaks at moiré reciprocal lattice vectors (*not* the reciprocal lattice vectors of the quadrupled unit cell, which are the m points of the moiré Brillouin zone). This reflects the more localized character of the second miniband's wavefunctions at smaller angles. We note that the ordinary structure factor $S(\boldsymbol{q}) = \frac{1}{N}\langle\rho(-\boldsymbol{q})\rho(\boldsymbol{q})\rangle$ (not shown) also does not show clear signatures of crystallization.

The origin of this behavior is as follows. The density operator projected to the nLL is $\bar{\rho}_n(\boldsymbol{q}) = P_{\mathrm{nLL}}\rho(\boldsymbol{q})P_{\mathrm{nLL}} = P_{\mathrm{nLL}}\sum_i e^{i\boldsymbol{q}\cdot\boldsymbol{r}_i}P_{\mathrm{nLL}} = L_n(\frac{\ell^2 q^2}{2})\tau_i(\boldsymbol{q})P_{\mathrm{nLL}}$ where $\tau_i(\boldsymbol{q})$ is a LL-index-independent magnetic translation operator acting on particle i (see, for instance, the Supplemental Material of Ref. [3]). Formally, one can map a many-body state in a given Landau level to

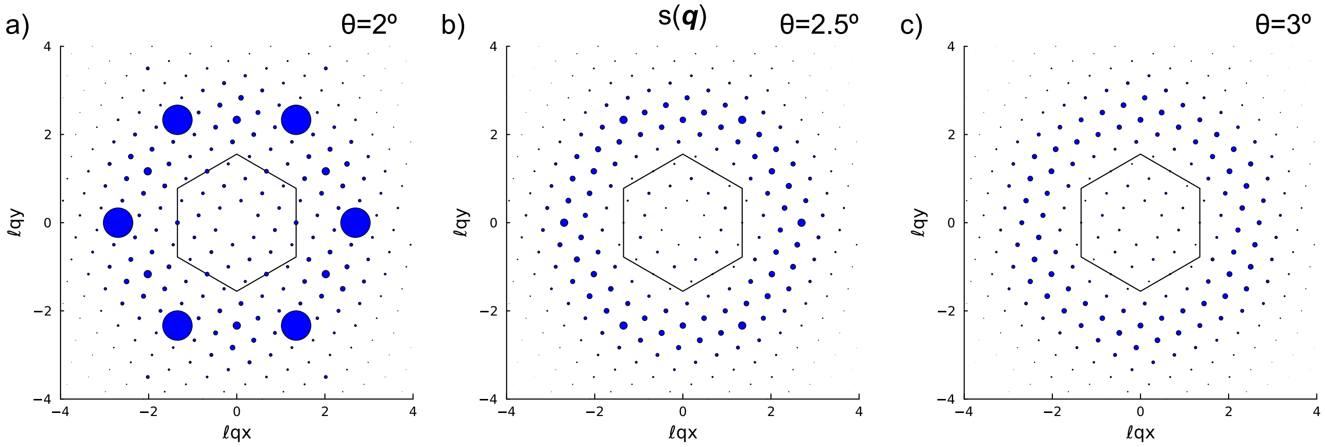

FIG. 3. **Projected structure factor at $n = \frac{3}{2}$ in the adiabatic model.** Projected structure factor $s(\boldsymbol{q}) = \frac{1}{N}\langle\bar{\rho}_2(-\boldsymbol{q})\bar{\rho}_2(\boldsymbol{q})\rangle$ of the ground state at $n = \frac{3}{2}$ in the adiabatic model at (a) $\theta = 2°$, (b) 2.5°, (c) 3°. Here $\langle\hat{O}\rangle = \frac{1}{N_{GS}}\sum_{i\in GS}\langle\Psi_i|\,\hat{O}\,|\Psi_i\rangle$ is an average over exactly degenerate ground states. Here $\bar{\rho}_2(\boldsymbol{q}) = \sum_{\boldsymbol{k}}\left\langle u_{2,\boldsymbol{k}-\boldsymbol{q}}\middle|u_{\boldsymbol{k}}\right\rangle c^{\dagger}_{2,\boldsymbol{k}-\boldsymbol{q}}c_{2,\boldsymbol{k}}$ is the projected density operator of the second miniband. Compare to the "modified" structure factor defined in the main text and shown in Fig. 1 of the main text. The first moiré Brillouin zone is drawn.

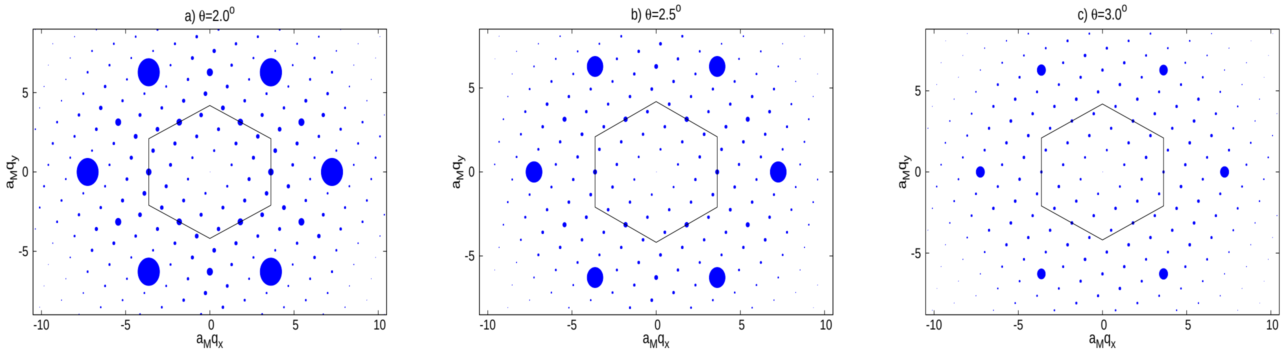

FIG. 4. **Projected structure factor at $n = \frac{3}{2}$ in the lowest-harmonic continuum model.** Projected structure factor $s(\boldsymbol{q}) = \frac{1}{N}\langle\bar{\rho}_2(-\boldsymbol{q})\bar{\rho}_2(\boldsymbol{q})\rangle$ of the ground state at $n = \frac{3}{2}$ in the lowest-harmonic model at (a) $\theta = 2°$, (b) 2.5°, (c) 3°. Here $\bar{\rho}_2(\boldsymbol{q})$ is the projected density operator of the second miniband. The first moiré Brillouin zone is drawn.

any other Landau level by acting with a product of Landau level index ladder operators. Under this mapping, the projected structure factor of the state in the nLL is related to that of the state in the LLL as $s_{n\mathrm{LL}}(\boldsymbol{q}) = \frac{1}{N}\langle\bar{\rho}_n(-\boldsymbol{q})\bar{\rho}_n(\boldsymbol{q})\rangle = \left[L_n(\frac{\ell^2q^2}{2})\right]^2 s_{\mathrm{LLL}}(\boldsymbol{q})$ where $L_n(x)$ is a Laguerre polynomial. Now $L_1(\frac{\ell^2q^2}{2} = 1) = 0$, which implies that $s_{1\mathrm{LL}}(q_0 \equiv \frac{\sqrt{2}}{\ell}) = 0$. Within the adiabatic model, the effective magnetic length ℓ and moiré lattice constant a_M are related by the property that the moiré unit cell encloses one effective flux quantum: $2\pi\ell^2 = \frac{\sqrt{3}}{2}a_M^2$. The magnitude of the m point wavevectors is then $|m| = \frac{2\pi}{\sqrt{3}}\frac{1}{a_M} = \sqrt{\frac{\pi}{\sqrt{3}}}\frac{1}{\ell} \approx 0.95q_0$. Therefore, when the second band is 1LL-like, suppression of finite size Bragg peaks at the m points is expected because $q = |m|$ is very close to a zero in $L_1(\frac{\ell^2q^2}{2})$. Specifically, $\left[L_1(\ell^2|m|^2/2)\right]^2 = s_{1\mathrm{LL}}(|m|)/s_{\mathrm{LLL}}(|m|) \approx 0.009$. Finally, Ref. [3] shows that the second miniband in the adiabatic model is indeed 1LL-like in a precise way, confirming the relevance of our LL-based argument to the adiabatic model.

In comparison, in Fig. 4, we show the projected structure factor $s(\boldsymbol{q})$ of the lowest-harmonic model's ground state at several twist angles. At $\theta = 2°$, $s(\boldsymbol{q})$ shows Bragg peaks at moiré reciprocal lattice vectors similar to the results of the adiabatic model. For the lowest-harmonic model, the $s(\boldsymbol{q})$ varies less with twist angles with no sharp peaks within the moirè Brillouin zone.

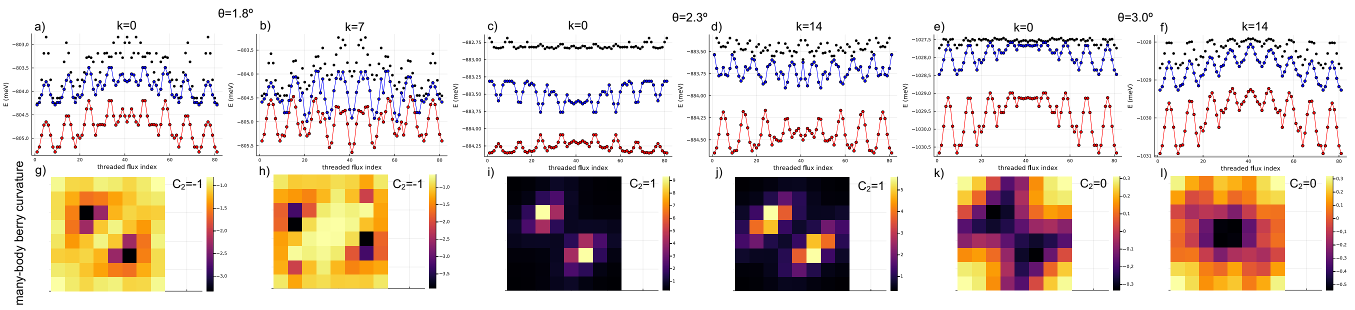

FIG. 5. **Many-body Berry curvature and Chern number of crystal states in the adiabatic model.** (a-f) Three lowest energy levels within the two non-symmetry-related center-of-mass momentum sectors as a function of threaded flux at several twist angles. The lowest is highlighted in red and the second lowest in blue, with lines added to guide the eye. (g-l) Many-body Berry curvature and Chern number at representative twist angles of the three distinct electronic crystals in the adiabatic model. $\epsilon = 5$. $n = \frac{3}{2}$. Cluster 28 is used. The many-body Berry curvature is in units such that its average is C_2.

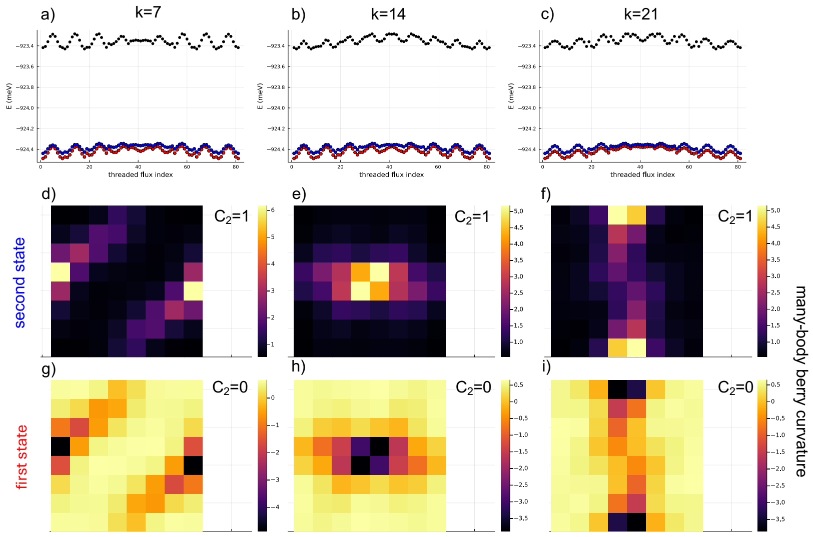

FIG. 6. **Many-body Berry curvature and Chern number of non-Abelian FCI in the adiabatic model.** (a-c) Lowest three energy levels in the three momentum sectors hosting the quasi-degenerate FCI ground states as a function of threaded flux. The lowest is highlighted in red and the second lowest in blue, with lines added to guide the eye. Many-body Berry curvature and Chern number of the second lowest energy state (d-f) and lowest energy state (g-i) in each ground state momentum sector. $n = \frac{3}{2}$. $\theta = 2.5°$, $\epsilon = 5$, and cluster 28 is used.

IV. MANY-BODY BERRY CURVATURE

We define the many-body Chern number as

$$C = 2\pi i \int_0^1 d\phi_1 \int_0^1 d\phi_2 \left[\left\langle \frac{\partial\Psi}{\partial\phi_1} \middle| \frac{\partial\Psi}{\partial\phi_2} \right\rangle - \left\langle \frac{\partial\Psi}{\partial\phi_2} \middle| \frac{\partial\Psi}{\partial\phi_1} \right\rangle \right]. \tag{1}$$

Here $|\Psi(\boldsymbol{\phi})\rangle$ is a many-body eigenstate of $H(\boldsymbol{\phi}) = U(\boldsymbol{\phi}) H U^\dagger(\boldsymbol{\phi})$ where $U(\boldsymbol{\phi}) = e^{i(\phi_1 \boldsymbol{T}_1 + \phi_2 \boldsymbol{T}_2)\cdot\sum_i \boldsymbol{r}_i}$. $\boldsymbol{L}_{1/2}$ are the primitive boundary vectors of the torus and $\boldsymbol{T}_a = \frac{2\pi \epsilon_{ab} \boldsymbol{L}_b \times \hat{z}}{|\boldsymbol{L}_1 \times \boldsymbol{L}_2|}$ are the primitive wavevectors.

Here we show the many-body Berry curvature and Chern numbers computed for degenerate or quasidegenerate ground states at various twist angles, elaborating on the results shown in Fig. 4 of the main text. In practice, we approximate the Berry curvature and Chern number by computing the discrete Berry flux through each plaquette of a discrete grid of threaded flux values (ϕ_i in Eq. 1) following the method described in Ref. [4]. This discrete method are generally reliable when the computed Berry flux through each plaquette, in units such that is average is the Chern number C, is much less than the number of plaquettes and varies smoothly between neighboring plaquettes. Specifically, we show C_2, the contribution to the total Chern number $C_{tot} = C_1 + C_2$ coming from the half-filled second band and its associated Berry flux. $C_1 = 1$ is the contribution from the full first band.

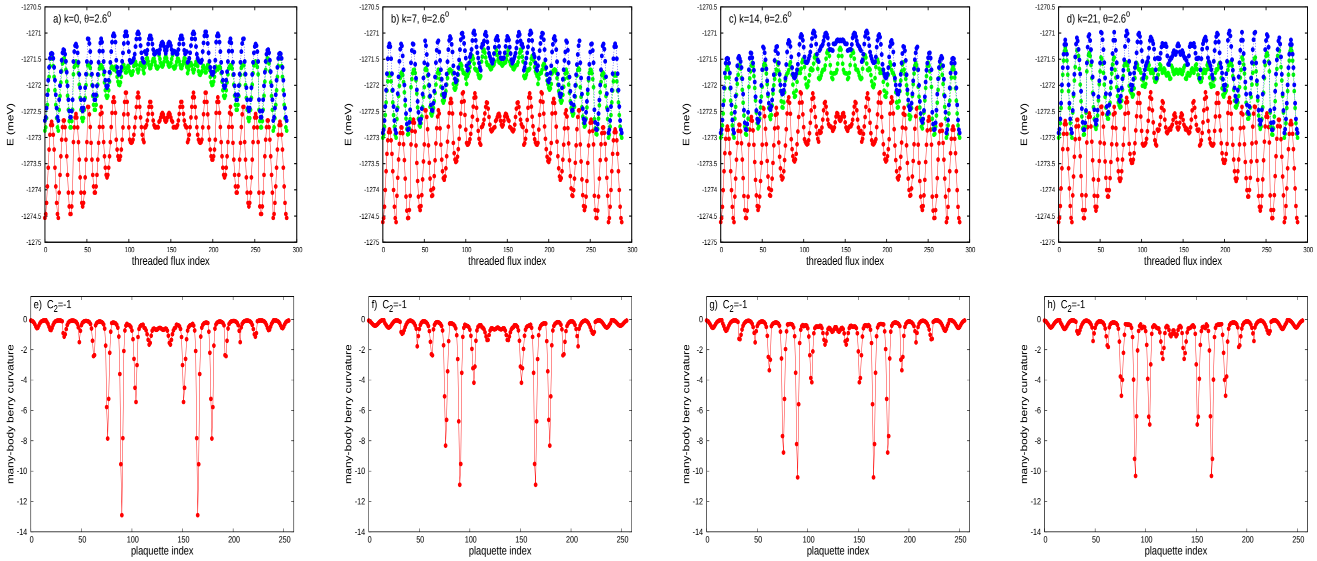

FIG. 7. **Many-body Berry curvature and Chern number of electron crystals in lowest-harmonic continuum model.** (a-d) The three lowest energy levels within each of the center-of-mass momentum quantum sectors k hosting quasi-degenerate crystal ground states as a function of threaded flux. The lowest is highlighted in red, the second lowest in green and the third lowest in blue, with lines added to guide the eye. (e-h) Many body Chern number and Berry curvature as a function of threaded flux. The many-body Berry curvature is in units such that its average is C. $\theta = 2.6$ and $\epsilon = 5$. Cluster 28 is used.

Fig. 5 shows data for the crystals and Fig. 6 shows data for the fractional Chern insulator. For each of the crystals, there is one ground state at each of the symmetry-related but inequivalent m points ($k = 7, 14, 21$ on cluster 28) and one at the γ point ($k = 0$ on cluster 28). We show data for the γ point ground state and for one representative of the three symmetry-related m points. In each case, the four quasidegenerate ground states have the same Chern number.

For the fractional Chern insulator, there are two degenerate ground states at each of the three inequivalent m points. At each m point, the Chern number of the lowest state is 0 and the Chern number of the second state is 1, as shown in Fig. 6. Therefore, the average Chern number of the sixfold degenerate ground state manifold is $C_2 = \frac{1}{2}$ as expected for a Pfaffian-like fractional Chern insulator.

We also show data for the crystal phase of the lowest-harmonic model Fig. 7. There is one ground state at each of the symmetry-related but inequivalent m points ($k = 7, 14, 21$ on cluster 28) and one at the γ point ($k = 0$ on cluster 28). We show data for these ground states at $\theta = 2.6$ and $\epsilon = 5$. In each case, the four quasidegenerate ground states have the same Chern number $C_2 = -1$ (we set the band Chern number as the reference $C_1 = 1$ and C_2 has the opposite sign to C_1 with $C_1 + C_2 = 0$).

V. MISCELLANEOUS

Fig. 8 shows an example many-body spectrum and modified structure factor a t $\theta = 2.3°$ in the adiabatic model.

Fig. 9 shows momentum-space diagrams of the finite-size clusters we use for exact diagonalization calculations in this work.

In Fig. 5(b) of the main text, the crystal momentum wavevector $\boldsymbol{k} = k_1 \boldsymbol{T}'_1 + k_2 \boldsymbol{T}'_2$ is assigned the k index $k_1 + N_1 k_2$ where $\boldsymbol{T}'_i = \boldsymbol{b}'_i / N_i$ and $N_1 = N_2 = 36$. $\boldsymbol{b}'_1 = \frac{2\pi}{\sqrt{3} a_M}(1, 0)$ and $\boldsymbol{b}'_2 = \frac{2\pi}{\sqrt{3} a_M}(-\frac{1}{2}, \frac{\sqrt{3}}{2})$ are primitive reciprocal lattice vectors of the quadrupled moiré unit cell.

[1] F. Wu, T. Lovorn, E. Tutuc, I. Martin, and A. MacDonald, Physical review letters **122**, 086402 (2019).
[2] A. P. Reddy, F. Alsallom, Y. Zhang, T. Devakul, and L. Fu, Phys. Rev. B **108**, 085117 (2023).
[3] A. P. Reddy, N. Paul, A. Abouelkomsan, and L. Fu, Phys. Rev. Lett. **133**, 166503 (2024).
[4] T. Fukui, Y. Hatsugai, and H. Suzuki, Journal of the Physical Society of Japan **74**, 1674 (2005).

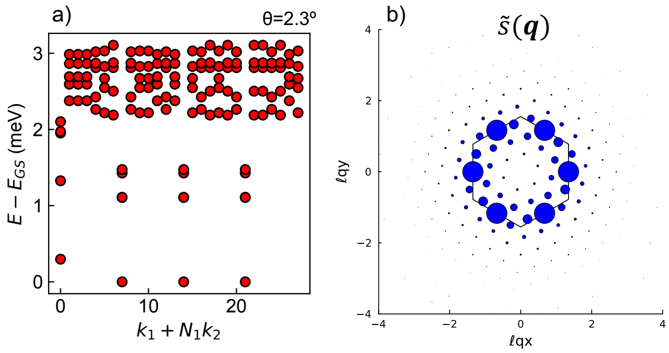

FIG. 8. **Crystal phase at $\theta = 2.3°$ in the adiabatic model.** (a) Many-body spectrum and (b) modified structure factor at $\theta = 2.3°$ in the adiabatic model. $\epsilon = 5$ and cluster 28 are used.

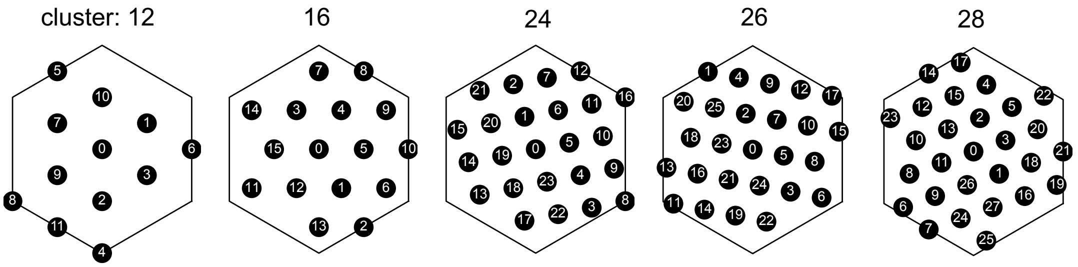

FIG. 9. **Finite-size cluster diagrams**. Momentum-space diagrams of finite-size clusters used in our exact diagonalization calculations. Each momentum point is labeled by an index $k = k_1 + N_1 k_2$.
